# Bi-Directional X-Ray Phase-Contrast Mammography

**DOI:** 10.1371/journal.pone.0093502

**Published:** 2014-05-13

**Authors:** Kai Scherer, Lorenz Birnbacher, Michael Chabior, Julia Herzen, Doris Mayr, Susanne Grandl, Anikó Sztrókay-Gaul, Karin Hellerhoff, Fabian Bamberg, Franz Pfeiffer

**Affiliations:** 1 Lehrstuhl für Biomedizinische Physik, Physik-Department & Institut für Medizintechnik, Technische Universität München, Garching, Germany; 2 Centre for Materials and Coastal Research, Helmholtz-Zentrum Geesthacht, Geesthacht, Germany; 3 Department of Pathology, Ludwig Maximilian University, Munich, Germany; 4 Department of Clinical Radiology, Ludwig Maximilian University, Munich, Germany; Wayne State University, United States of America

## Abstract

Phase-contrast x-ray imaging is a promising improvement of conventional absorption-based mammography for early tumor detection. This potential has been demonstrated recently, utilizing structured gratings to obtain differential phase and dark-field scattering images. However, the inherently anisotropic imaging sensitivity of the proposed mono-directional approach yields only insufficient diagnostic information, and has low diagnostic sensitivity to highly oriented structures. To overcome these limitations, we present a two-directional x-ray phase-contrast mammography approach and demonstrate its advantages by applying it to a freshly dissected, cancerous mastectomy breast specimen. We illustrate that the two-directional scanning procedure overcomes the insufficient diagnostic value of a single scan, and reliably detects tumor structures, independently from their orientation within the breast. Our results indicate the indispensable diagnostic necessity and benefit of a multi-directional approach for x-ray phase-contrast mammography.

## Introduction

Despite the introduction of extensive screening programs in the 80's and a rapid promotion of mammography systems, breast cancer is still the second most common cause of cancer death for women worldwide [1]. The most prevailing difficulties of modern diagnostic mammography are a non-negligible number of false-positive diagnoses, accompanied by redundant medical follow-up (10.7%) and a relatively high rate of undetected tumors (21.9%), most prevalent in the examination of younger women [2]. Especially, dense breast soft-tissue exhibits only minor deviations with respect to attenuation characteristics, resulting in a poor imaging contrast for conventional mammography. Phase-sensitive x-ray imaging methods show the potential to circumvent this problem by utilizing the intrinsically enhanced electron density contrast [3].

Initial mammography studies confirming diagnostic benefits of the superior soft-tissue contrast of phase-sensitive techniques were carried out with synchrotron radiation [4], [5]. The introduction of so called Talbot-Lau interferometers in combination with a conventional laboratory x-ray source pushed phase-contrast imaging towards possible clinical applications [6]. In grating-based differential phase-contrast (DPC) imaging, phase stepping enables the simultaneous retrieval of differential phase, dark-field and conventional absorption signal [6]–[8]. The differential phase signal is obtained by measuring the angular deviations of the transmitted wavefront, which are directly linked to the local gradient of the tissue's phase shift. Recent differential phase-contrast mammography studies conducted with freshly dissected complete breasts showed promising results with respect to clinical relevance and feasibility [9].

However, previous efforts also indicated that a direct translation of radiographic DPC towards conventional mammography is accompanied by two major drawbacks: First, the retrieved local phase gradient is of differential nature, which results in strongly edge-enhanced images with a reduced visual perception of extended structures. One-dimensional integration of the differential data is possible, however requires the implementation of regularization algorithms in order to reduce streak artifacts in direction of the integration (accumulation of statistical errors) affecting the integrated phase [10], [11]. Second, since the gratings are only structured along one dimension, DPC imaging sensitivity is highly anisotropic, being only non-zero perpendicular to the grating lines. More precisely, only the component of electron density changes perpendicular to the grating lines can be accessed and contributes to the differential phase-contrast signal. Similarly, tissue-induced small-angle scatter, which determines the dark-field signal, remains undetected if emitted parallel to the grating bars [12]. Consequentially, the risk to overlook oriented, non-uniform structures within the breast such as ducts and tumor branches is high.

In this work, we apply a bi-directional imaging approach, composed of two orthogonal scans, to ensure orientation sensitivity while remaining in a radiographic acquisition mode [13]–[15]. We investigate the clinical relevance of this method by measuring an invasive ductal carcinoma (IDC) enclosed within the freshly dissected sample. By means of this medically significant tumor which comprises 72.8% of all invasive breast cancer we subsequently prove the necessity of an isotropic detection sensitivity [16]. We evaluate the diagnostic advantages of two-dimensional integrated phase compared to absorption imaging. Furthermore, insights into the tumor morphology are obtained by analysing angular deviations in the scattering signal (directional dark-field imaging) [12], [17]. The conclusion of our study is ascertained by a histological verification of our findings.

## Materials and Methods

The study was conducted in accordance with the Declaration of Helsinki and was approved by the local ethics committee (Ethikkommission of the Ludwig-Maximilian-University, Munich). Inclusion criteria were indication for surgical removal of a benign or malignant breast tumor after previous core biopsy. Patients had no previous hormonal therapy or chemotherapy. Participants gave written consent before participation after adequate explanation of the study protocol. Indication for breast surgery followed recommendation of the interdisciplinary tumor board of the University of Munich breast center.

**Figure 1:**
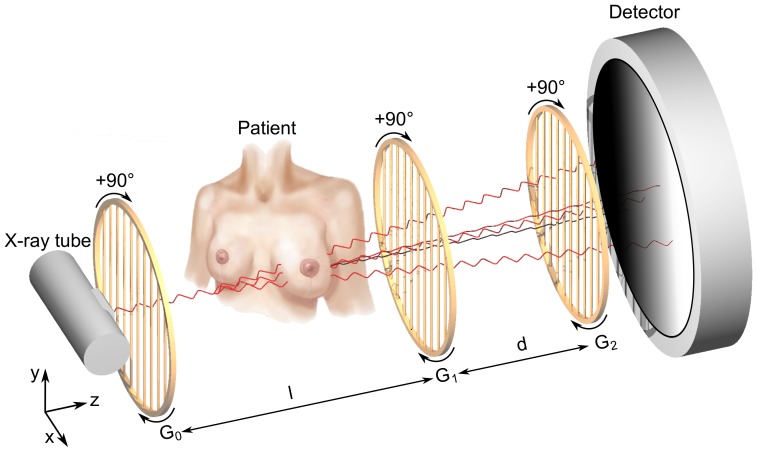
Schematic overview of the bi-directional phase-contrast mammography setup. The setup consists of a conventional x-ray tube, a source grating *G*_0_, a phase grating *G*_1_, an analyser grating *G*_2_ and a flat panel detector. The two-directional, orthogonal scanning approach is indicated by a 90° rotation of the gratings. For practical reasons the rotation of the specimen was preferred.

The measurements were conducted with a three-grating Talbot-Lau interferometer (Fig. 1), consisting of a conventional x-ray tube, a source grating 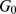, a phase grating 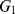 (design energy of 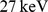), and an analyser grating 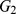 (obtained from Mikroworks GmbH, Germany). A compact layout in the third Talbot order (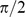-shift) was chosen, with distances 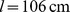 between 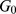 and 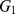 and 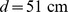 between 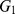 and 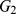, respectively. The corresponding grating periods were 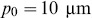, 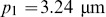 and 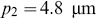. A Varian Paxscan 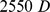 flat panel with Gadox screen (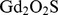) and 127×127 mm-pixel size was used as detector. The Nonius FR 591 rotating anode x-ray generator with a molybdenum target was set to 40 kVp/70 mA. The sample was positioned at 2.7 cm directly upstream 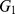 in order to optimize the sensitivity of the interferometer [18].

**Figure 2:**
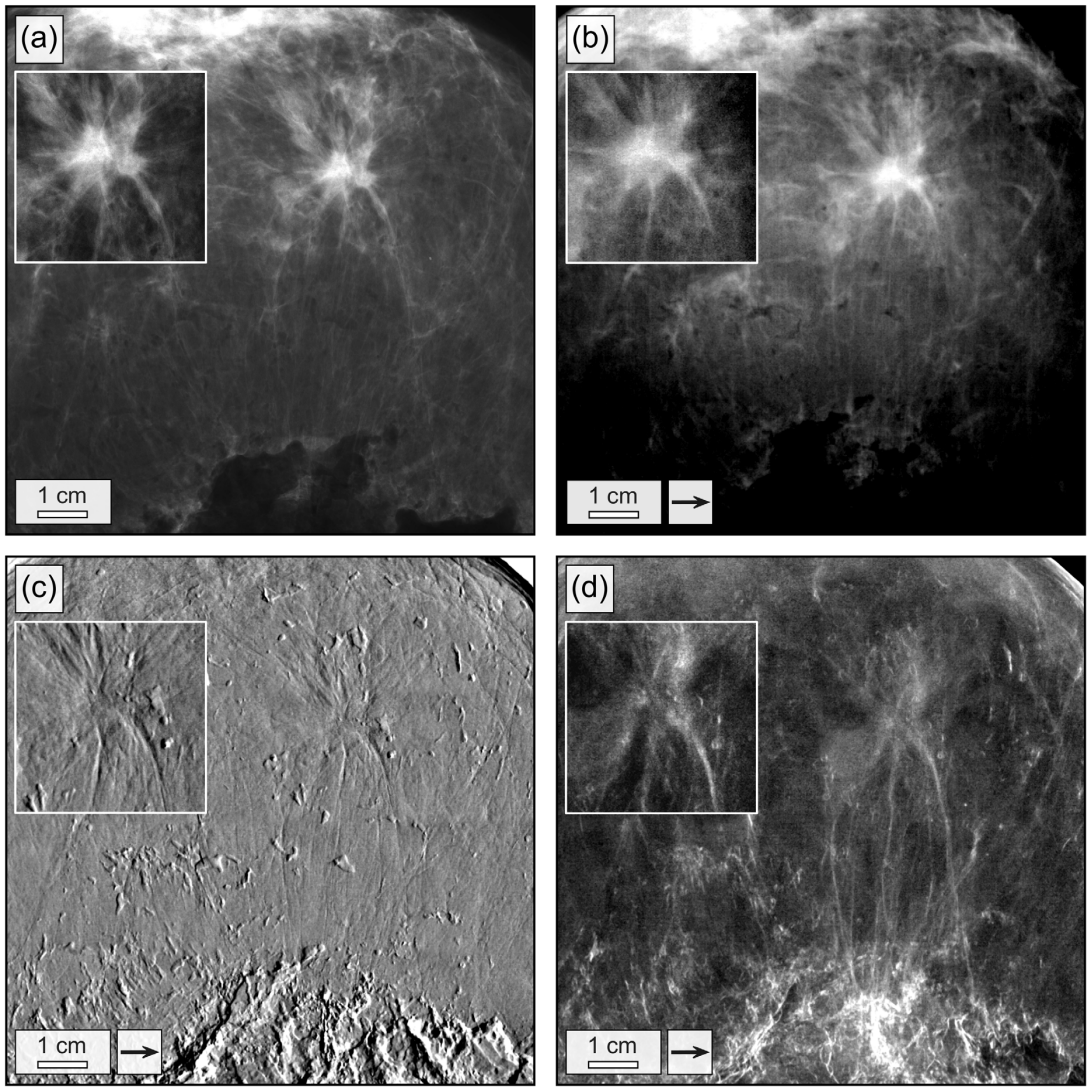
Conventional and grating-based mammograms of a breast specimen with invasive ductal carcinoma. Conventional mammogram (a), grating-based absorption (b), differential phase (c) and dark-field image (d) of the breast specimen. The field of view has a size of 12.8×12.8 cm^2^. Images (b)–(d) were obtained by stitching together 4×4 low-statistic projections. Arrows indicate the direction of scanning. The high-statistic images of the invasive ductal carcinoma are shown as inlays.

The imaging of the freshly dissected mastectomy specimen was performed in craniocaudal orientation. For reasons of comparability and benchmarking, a digital mammogram (29 kVp, 120 mA, average glandular dose 1.22 mGy) of the specimen within the sample holder was conducted using a Selenia Dimensions scanner from Hologic (Fig. 2a). The sample holder consisted of two 4 mm thin polycarbonate plates bordered in a metal frame to apply a medically reasonable sample compression of 5 cm, while minimizing the reduction of flux by the attenuation.

Firstly, to identify the tumorous region, one image of the complete breast was taken with the interferometer. A total field of view of 12.8×12.8 cm^2^ was realized by stitching 4×4 projections. Scans with 9 phase steps and an exposure time of 9 seconds per step (low-statistic) were performed with phase stepping in *x*-direction (Fig. 1). Hence, underlying imaging sensitivity was strongly anisotropic and only components of the signals in *x*-direction detected. Only one flat-field, e.g. projection without the sample in the beam, was taken beforehand. A smooth stitching of the 16 projections was achieved by correcting drift-related offsets within the differential phase. This was done by leveling the overall DPC signal of adjacent projections within the region of joint overlap. The processed and stitched images (Fig. 2) related to absorption (b), differential phase (c) and dark-field (d) were obtained by Fourier analysis [6].

On the basis of the complete DPC breast images (Fig. 2b–d), the position of the tumorous area was determined, by which two-directional differential phase-contrast mammography is showcased in the following: An isotropic DPC imaging sensitivity is realized by combining the two one-dimensional and mutually orthogonal measurements of the IDC with phase stepping in *x* and *y*-direction, respectively. Two high statistic scans were performed with 13 phase steps and an integration-time of 13 seconds per step each, in order to be able to exclude statistical error as the cause of the presented results. The inlays in Figure 2 show the corresponding images of the IDC acquired with phase stepping in *x*-direction. The thereto orthogonal scan in *y*-direction was performed by rotating the sample holder by 90° and then relocating the sample stage to preferably image the same sample volume. For the purpose of demonstrating bi-directional phase-contrast mammography, latter was preferred over the rotation of all three gratings by 90°. Alignment and compression of the breast tissue remain unaltered within the sample holder during the measurement. In the following, images retrieved along one dimension are marked by a subscript “*x*” or “*y*”, respectively.

Thereafter, the high-statistic set of absorption (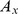, 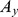), differential phase (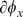, 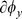) and dark-field images (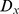, 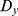) of the IDC were fused. Firstly, it had to be ensured that the two images of each modality cover precisely the sample section. A possible displacement within the three image sets was reduced by using a least square optimization algorithm, which shifts the two absorption images against each other until the overall discrepancy in intensity between both images is minimized. This is feasible, since the detection of wave attenuation (absorption image) in comparison to wave deviations (differential phase, dark-field image), is independent from the scanning direction. The numerically determined shift (subpixel accuracy) was then used to correct for the displacement within the set of absorption, differential phase and dark-field images.

The fusion of the one-dimensional phase gradients 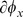 and 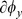 was realized via a two-dimensional Fourier transform approach [15]. The two-directional integrated absolute phase 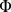 is then given by
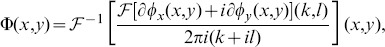
(1)where 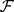 is the two-dimensional Fourier transform, 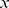, 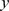 the Cartesian and 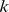, 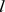 the reciprocal space coordinates. The combined image 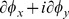 was mirrored horizontally and vertically to avoid edge discontinuities at the image boundaries, thus providing periodic data in Fourier space [19]. Since the phase integration corresponds to a 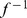-filter (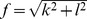) in Fourier space, low frequencies are enhanced compared to high frequencies. The amplification of low frequencies generates a background, that is superimposed on the integrated phase image 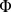. The corrected phase image 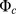 is obtained by
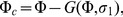
(2)where 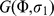 is the integrated phase image 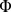, blurred with a Gaussian filter of width 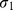. By intensively blurring (standard deviation of Gaussian kernel of 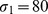, image size of 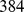
×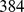 pixels) relevant image contents remain unaffected by the subtraction of the low frequency background.

**Figure 3:**
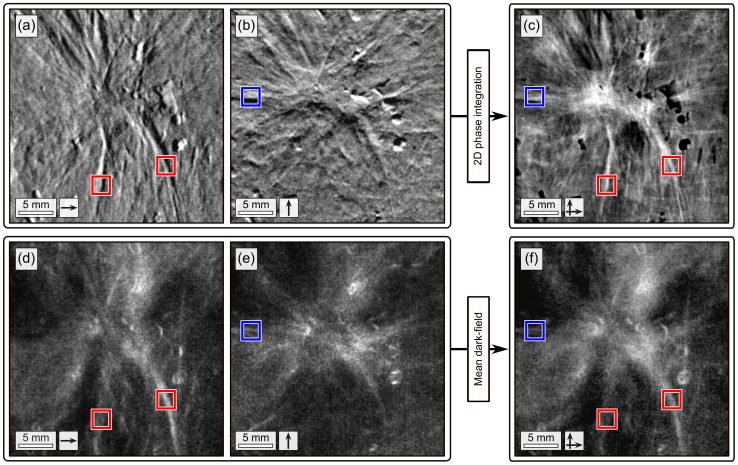
Two-directional, grating-based mammograms of the invasive ductal carcinoma. Two-directional differential phase 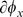 (a), 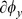 (b) and sharpened, two-dimensional integrated phase image 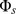 (c). Dark-field 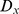 (d), 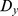 (e) and mean dark-field 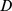 image (f). Arrows indicate the direction of scanning. Red and blue boxes indicate tumor branches exclusively perceivable in the images obtained with scanning performed in 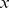
- or 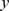-direction, respectively.

The noise power spectrum of the input images 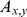, 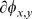 and 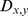 is approximately inversely proportional to 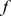 [20]. Due to integration, the retrieved phase image exhibits a noise power spectrum proportional to 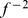. The low amplitude of high frequency noise in the image allows a high frequency boost of 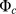, in order to pronounce small tumor features and increase image acutance. The sharpened phase image 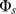 (Fig. 3c) is obtained with an unsharp mask operation, given by
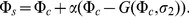
(3)


To pronounce very fine structures, a slight blur (standard deviation of Gaussian kernel of 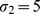, image size of 384×384 pixels) was used. The filter strength was set to 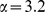.

The dose-equivalent absorption image is calculated by
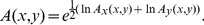
(4)


**Figure 4:**
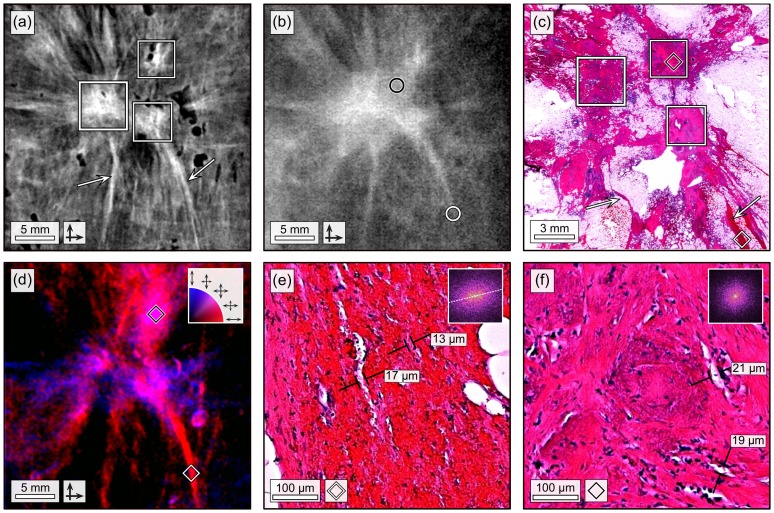
Two-directional, grating-based mammograms of the invasive ductal carcinoma with correlated histopathology. (a) Sharpened, two-dimensional integrated phase image 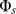 of the invasive ductal carcinoma. The frames in (a) and (c) indicate three locally separated tumor lesions (apparently trifocal). Arrows in (a) and (c) indicate fine tumor branches. (b) Sharpened, absorption image 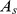 of the invasive ductal carcinoma. Circles indicate position of relevant tumor details. (c) Histological slice (Hematoxylin and Eosin stain) of the invasive ductal carcinoma. (d) Directional dark-field image of the invasive ductal carcinoma. Preferred scattering direction is color-coded ranging from 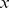-directed (red) over isotropic (purple) to 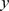-directed (blue). (e) 200x magnified histological image of the tumor branch, as indicated by white diamond in (c) and (d). The 2-dimensional FFT is shown as an inlay. (d) 200x magnified histological image of the tumor lesion, as indicated by black diamond in (c) and (d). The 2-dimensional FFT is shown as an inlay.

To provide comparability to the sharpened phase image 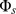, the absorption image 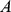 was also processed with the unsharp mask filter (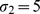). However, in case of the absorption image 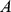, the noise power spectrum is proportional to 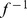. This allows sharpening only to a minor extent (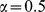), until the intrinsic amplification of high frequency noise leads to a degradation of image quality [21]. The sharpened absorption image 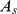 is shown in Figure 4b.

The mean dark-field image 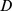 (Fig. 3f), related to the overall small angle scattering power of the sample, is given by
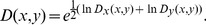
(5)


Moreover, the dark-field images 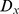 and 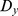 were utilized to obtain information on the preferred scattering direction of the tissue under investigation. Scattering in x-direction (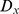) is colored red, while scattering in y-direction (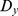) is colored blue. The RGB-composition (Fig. 4c) of the two images, classifies scattering power (brightness) and the preferred scattering direction ranging from *x*-directed (red) over isotropic (purple) to *y*-directed (blue).

The applied radiation dose was measured using the incident air kerma with a Dosimax plus/RQX-detector system. The incident air kerma rate was found to be 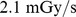 at the beam center. For the initial 4×4 low-statistic projections, 9 phase steps with an integration-time of 9 seconds each were taken. In case of the high-statistic projections used for the two-directional proof of principle study the number of steps and integration-time was increased to 13. To calculate the mean glandular dose a Monte-Carlo based conversion factor of 0.389 was chosen, determined by the half-value layer (Al) of 0.8 mm and a sample thickness of 5 cm [22], [23]. A mean glandular dose of 66 mSv (total exposure time of 81 seconds, low-statistic) and 138 mSv (total exposure time of 169 seconds, high-statistic) per projection was found. While we acknowledge that these values exceed the dose limits set for clinical mammography, several factors can be addressed to achieve dose-compatible measurements in near future (see discussion).

## Results

An overview of the high-statistic differential phase and dark-field images of the IDC, as obtained with the two-directional scanning approach is given in Figure 3. We investigate the dependency between the detection of strongly oriented tumor features and scanning direction, by comparing 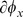 against 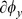 and 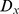 against 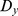, respectively: The tumor branches that are primarily vertically aligned (red framed) are strongly visible in 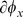 (Fig. 3a) and 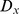 (Fig. 3d). However, the same features cannot be detected in the thereto orthogonal images 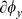 (Fig. 3b) and 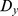 (Fig. 3e). By contrast the horizontally oriented tumor branch (blue framed), is exclusively detected in 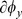 (Fig. 3b) and 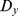 (Fig. 3e).

These observations indicate, that tumor branches exhibit a strongly anisotropic contrast for both differential phase and dark-field imaging: Within the tumor branches, electron density variations of the tissue are only minor, resulting in a poor differential phase contrast along the branch. However, compared to the surrounding, healthy tissue, the electron density is differing considerably. Furthermore, scatter caused by the branches and its substructures must be directed mostly perpendicular to the branch orientation.

Hence, the selection of scan direction, and therewith one-dimensional imaging sensitivity, determines to what extent the anisotropic tissue structures like branches, embedded in healthy tissue, can be detected. If scanning is performed parallel to the branch orientation for instance, neither scatter, caused by the branches, is detected, nor sufficient phase-contrast obtained. To secure a full detection of the tumor branches, an isotropic imaging sensitivity is indispensable, as provided by the combination of two orthogonally conducted scans. Comprehensive images, that provide the full range of features (red and blue frames), are obtained by fusing the one-directional differential phase and dark-field images as sharpened, two-directional integrated phase 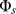 (Fig. 3c) and mean dark-field 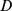 (Fig. 3f), respectively. Moreover, the sharpened, two-directional integrated phase image 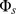 is free of streak and shadowing artifacts, even though the field of view is exceeded by the sample.

Besides overcoming the limited sensitivity of a one-directional scan, the two-directional approach exhibits further diagnostic advantages, illustrated in a benchmark (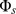 vs. 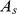) and directional dark-field case study (Fig. 4). The sharpened phase image 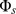 (Fig. 4a) enables a diagnostic differentiation of the carcinoma into three locally separated tumor lesions (white frame). Two tumor branches are clearly perceivable, as directly emerging from the single lesions and spreading out to the image borders (arrows). The superior depiction of fine details within the phase image is caused by an enhanced soft-tissue contrast, but also improved local contrast and image acutance gained by image sharpening. By contrast, the same medical details can not be deduced from the dose-equivalent, sharpened absorption image 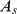 (Fig. 4b): The attenuation-based soft-tissue contrast is insufficient to enable a visual distinction into lesions, but instead displays the tumor mass as pervasive structure (black circle). The tumor branches appear distinctively shortened, since fine branch parts (high frequency features) are blurred within image noise (white circled). To validate the medical relevance of details exclusively perceivable within the phase image and exclude them as being image artifacts, histological evaluations were carried out. The tumorous area was extracted, dissected and stained with Hematoxylin and Eosin (Fig. 4c). We matched the three tumor lesions (white frame) and fine branches (arrows) with respect to size and orientation and confirmed their malignancy. Furthermore, the tumor was classified as apparently trifocal, e.g. containing three locally separated lesions.

We extracted further diagnostic insights on the tumor constitution by examining two spots of the directional dark-field image (Fig. 4d) in more detail. The vertically oriented tumor branch (white diamond) scatters preferably perpendicular to its orientation, identifiable by the red color. This prominent anisotropic scatter can be ascribed to the tissue morphology on the scatter determining length scale, which is in the order of the analyser grating period of 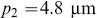 [24]. The corresponding histological section of the branch was photographed with a magnification of 200 (Fig. 4e). The magnified image reveals multiple vascular walls (white) and elongated tumor cells (pink) preferably aligned alongside the fibre with diameters in the range of some microns. The elliptically-shaped two dimensional Fourier transform of the image (top right inlay) emphasizes the inherent alignment of the substructure. Since scatter occurs dominantly at edges/tissue-interfaces which are mutually oriented along the fibre, an unilaterally directed scatter is generated.

For a second correlation of directional dark-field with tumor morphology, the top tumor lesion (black diamond) was chosen. Here contributed scatter is uniformly distributed, discernible by the violet color. The corresponding histological section of the branch was photographed with a magnification of 200 (Fig. 4f). The magnified image shows a cluster of tumor cells (violet) surrounded by bunches of elongated cells (pink) and vascular walls (white). By contrast, in this case the morphology seems random, without exhibiting any preferred orientation or distinct alignment of the inherent features. This impression is ascertained by the circular-shaped two-dimensional Fourier transform of the image (top right inlay). Accordingly, scatter in this area is isotropic.

## Discussion

In this study, we demonstrated that grating-based mammography faces the problem of a diagnostically limiting imaging sensitivity, if operated with an mono-directional scan only. We identified structures associated with an IDC, that exhibit a strongly anisotropic imaging contrast for both differential phase and dark-field imaging. To provide diagnostic reliability and ensure detection of oriented, tumorous structures, regardless their orientation within the breast, an isotropic imaging sensitivity is indispensable and could be provided by applying a two-directional scanning approach. An isotropic imaging sensitivity is crucial requirement for the successful detection of early stage tumors such as in-situ carcinoma. Here tumor growth is naturally limited to the inner of ducts, resulting in a elongated tumor shape similar to the observed IDC branches [25], [26].

We have proven feasibility of the two-directional scanning and phase-integration approach by first localizing the tumor and then subsequently measuring the corresponding enclosed section within the undissected mastectomy specimen.

To exclude statistical errors as cause of the observed anisotropic imaging contrast given by IDC branches, this proof of principle study had to be conducted with high-statistic measurements and thus should neither be considered dose nor time relevantly designed. Stampanoni et al. [9] and Sztrókay et al. [27] already discussed that an acquisition of images within the dose limits required for clinical applications would be possible by optimizing several setup relevant aspects such as design energy, x-ray target material, beam filtration, grating quality, duty-cycle, grating substrate thickness, sample holder and detector efficiency. An increase of currently 13.2% to 49.5% setup fringe visibility by improving beam and grating quality would imply an approximately 14-fold decrease in necessary dose for DPC imaging. Further we expect that an optimization of the detector with respect to the design energy of 27 keV, the replacement of the x-ray target molybdenum with tungsten and the exclusion of polycarbonate plates would double the setup efficiency. These improvements could decrease the current dose of 66 mSv per projection down to 2.4 mSv which is within the dose limits of 2.5 mSv set by the European guidelines for quality assurance in breast cancer screening and diagnosis [28].

We elaborated additional diagnostic advantages accompanied by the two-directional scanning approach which could further motivate the need of acquiring two images: The two-dimensional integration mechanism provides absolute, streak artifact free phase images which proved to facilitate the visual differentiation of medically significant tumor details, yet unseen in the dose equivalent absorption image. The excellent depiction of high frequency features yields the potential to strongly promote the detection of early stage tumors, fine tumor branches and small metastases.

Furthermore, we examined a possible diagnostic application of directional dark-field imaging by characterizing the soft-tissue morphology on the micrometer length scale, far below the actual detector resolution. The morphology of two tumor associated areas could be characterized by their scattering behavior as randomly proliferated and inherently oriented, respectively. This technique may promote the characterization and therewith diagnostic classification of suspicious structures and microcalcifications as linear branching or coarse granular [29], [30]. Besides, it might give a hint concerning the neoplastic grading of tumorous tissue by distinguishing between well-differentiated/oriented and fully anaplastic cell domains.

Finally, the 90° rotation of the three gratings could be circumvented by the implementation of two-dimensional structured gratings [13].
